# Factors affecting emergency medical dispatchers decision making in stroke calls – a qualitative study

**DOI:** 10.1186/s12873-024-01129-0

**Published:** 2024-11-15

**Authors:** Bjørn Jamtli, Edel Jannecke Svendsen, Trine Møgster Jørgensen, Jo Kramer-Johansen, Maren Ranhoff Hov, Camilla Hardeland

**Affiliations:** 1 Faculty of Health Sciences, Oslo Metropolitan University, Oslo, Norway; 2 Division of Prehospital Services, Oslo University Hospital, Oslo, Norway; 3 Faculty of Health, Welfare and Organisation, Østfold University College, Fredrikstad, Norway; 4 Norwegian National Advisory Unit on Prehospital Emergency Medicine, Oslo University Hospital, Oslo, Norway; 5 Air Ambulance Department, Oslo University Hospital, Oslo, Norway; 6 Institute of Clinical Medicine, University of Oslo, Oslo, Norway; 7 Department of Neurology, Oslo University Hospital, Oslo, Norway; 8 The Norwegian Air Ambulance Foundation, Oslo, Norway; 9 Department of Research, Sunnaas Rehabilitation Hospital, Bjørnemyr, Norway

**Keywords:** Prehospital care, Emergency medical services, Emergency calls, Emergency medical dispatch, Emergency Medical Communication Center, Prehospital stroke management, Stroke pathway, Triage.

## Abstract

**Objectives:**

Emergency Medical Communication Centers (EMCC) have a key role in the prehospital chain-of-stroke-survival by recognizing stroke patients and reducing prehospital delay. However, studies on EMCC stroke recognition report both substantial undertriage and overtriage. Since mis-triage at the EMCC challenges the whole chain-of-stroke-survival, by occupying limited resources for non-stroke patients or failing to recognize the true stroke patients, there is a need to achieve a more comprehensive understanding of the dispatchers’ routines and experiences. The aim of this study was to explore factors affecting EMCC dispatcher’s decision-making in stroke calls.

**Materials and methods:**

A qualitative exploratory study, based on individual semi-structured interviews of 15 medical dispatchers from EMCC Oslo, Norway. Interviews were conducted during August and October 2022 and analyzed using the principles of thematic analysis.

**Results:**

We identified four themes: [1] Pronounced stroke symptoms are easy to identify [2]. Non-specific neurological symptoms raise suspicion of acute stroke but are difficult to differentiate from other medical conditions [3]. Consistent use of the Criteria Based Dispatch (CBD) protocol may increase EMCC overtriage [4]. Contextual conditions at EMCC can affect dispatchers’ decision-making process and the ability for experiential learning.

**Conclusions:**

Medical dispatchers at the EMCC perceive vague and non-specific stroke symptoms, such as dizziness, confusion or altered behaviour, challenging to differentiate from symptoms of other less time-critical medical conditions. They also perceive the current CBD protocol in use as less supportive in assessing such symptoms. High workload and strict EMCC response time interval requirements hinder the gathering of essential patient information and the ability to seek guidance in cases of doubt, potentially exacerbating both EMCC undertriage and overtriage. The absence of feedback loops and other strategies for experiential learning in the EMCC hampers the medical dispatcher’s ability to evaluate their own assessments and improve dispatch accuracy.

**Supplementary Information:**

The online version contains supplementary material available at 10.1186/s12873-024-01129-0.

## Background

The primary task of Emergency Medical Communication Centers (EMCC) is to assess emergency calls, identify the nature of the medical conditions and determine the appropriate medical response and priority [1]. Assessments and decisions regarding emergency calls at EMCCs are considered a multifaceted interactive task, involving the gathering, processing and evaluation of patient information based on the dialogue with the caller within a limited time frame [2].

From other studies, we know that the EMCCs serve as the initial point of contact for most patients experiencing stroke symptoms and have a key role in the prehospital-chain-of-stroke-survival [3–5]. Nevertheless, recognizing stroke patients at EMCC poses a particular challenge for dispatchers due to the inability to visually observe and physically examine the patient [2, 6]. Moreover, stroke symptoms can be vague and non-specific and other medical conditions can mimic stroke symptoms [7–11].

EMCC undertriage in acute stroke could be defined as stroke patients not identified by medical dispatchers at the EMCC, subsequently receiving low-priority medical response [12]. As a result, undertriage could increase patient delays, consequently reducing treatment rates and worsening outcomes [13, 14]. Conversely, excessive stroke dispatches to non-stroke patients defined as EMCC over-triage, could consume limited pre- and in-hospital resources, and result in the under-prioritization of other medical emergencies [15, 16]. As a consequence, quality of EMCC stroke assessments should be evaluated not only based on the ability to recognize acute stroke patients, but also to rule out such conditions. These challenges are increasingly pertinent for all Emergency Medical Services (EMS) due to the ageing population with increased consumption of healthcare services [17, 18], but also the overall increased demand for ambulance services, and particularly, the extended increase in the number of high-priority ambulance missions [19–21].

A systematic review on EMCC stroke recognition published in 2024 revealed substantial variations in stroke sensitivity, ranging from 17.9 to 83%, with corresponding positive predictive values (PPV) ranging from 24 to 87.7% [22]. The results also align with the results from our previous study from EMCC Oslo, Norway, which revealed a stroke sensitivity of 77% and a PPV of 16% [23]. These variations suggest both substantial EMCC undertriage and overtriage. Despite these findings, no obvious trend towards improvement in EMCC stroke sensitivity or positive predictive value (PPV) has been identified during the last decades [22]. The absence of improvement may be attributed to a limited understanding of the underlying reasons for these variations. This may stem from sparse research regarding improvement in emergency call handling in general, but also the predominant use of quantitative research methods and limited focus on how contextual conditions could affect medical dispatcher’s assessments [2, 24–26].

The aim of this study was to explore factors affecting dispatcher decision-making in stroke calls, subsequently impacting stroke dispatch accuracy, using a qualitative approach.

## Methods

The study had a qualitative exploratory design, based on individual semi-structured interviews of emergency medical dispatchers from EMCC Oslo, Norway. The design was chosen to obtain, interpret and summarize experiences and practices from EMDs, as well as to achieve a comprehensive understanding of their views [27, 28]. The study is part of the research project; The Dispatch –Norwegian Acute Stroke Prehospital Project (NASPP), aiming to improve EMCC stroke recognition.

### Setting

EMCC Oslo is located at Oslo University Hospital Ullevål and serves a population of approximately 1.7 million inhabitants in the southeastern part of Norway. The EMCC handles more than 260.000 emergency calls annually and coordinates approximately 70 ground ambulances and 5 Helicopter Emergency Medical Services (HEMS).

Although Norway has implemented the common emergency number 112 for the European Union (EU), it also maintains a dedicated toll-free public emergency medical number 113 which is routed to one of the 16 EMCCs in Norway. The medical emergency number 113 is the preferred point of contact for the public in cases of acute and serious illnesses or injuries, handling nearly 780.000 calls annually [29]. According to a separate regulation of prehospital emergency medical services in Norway [30], all calls to the emergency medical number 113, are answered directly by EMCC dispatchers who are specially trained nurses or paramedics without any pre-screening. The regulation also stipulates that 90% of all inquiries to the emergency medical number 113 should be answered within 10 seconds.

Dispatch assessments and triage at EMCC in Norway rely on the dispatcher’s medical knowledge and their use of “The Norwegian Index for Emergency Medical Assistance” (Index), a Criteria-Based dispatch (CBD) decision support tool [31]. This Index comprises a Start page and 39 different symptom-based criteria cards. Most of the criteria cards contain lists of symptoms, corresponding responses and priorities, as well as additional information about symptoms and potential causes. Dispatch priorities are categorized as red (acute and life-threatening conditions), yellow (urgent and potentially life-threatening conditions), and green (non-urgent conditions). After selecting a criteria card, dispatchers assess the criteria from top (the most acute criteria) to bottom (non-urgent criteria) until one criterion is met. Stroke criteria are listed in criteria card 27- *Altered levels of consciousness/paralysis*, and criteria card 39 – *Eyes*. The criteria cards include both pronounced stroke symptoms, such as sudden speech disturbance, facial weakness, or unilateral weakness in an arm or leg, consistent with the Face-Arm-Speech-Time (FAST) test, but also non-specific stroke symptoms such as increasingly confused or drowsy, sudden intense headache and tendency to faint [32]. The dispatch protocol *Start page*, criteria card 27 and criteria card 39 – *Eyes*, are available in Supplemental materials.

If patient symptoms are identified as suspected stroke symptoms by the EMCC dispatcher, the default response will be to dispatch the nearest ambulance with a stroke criteria and acute priority (lights and sirens). If the call is triaged as a low-acuity call (non-stroke), the call will be transferred to the local Out- of- hours general practitioner (GP) acute clinic, or family GP for further evaluation. Patients with suspected stroke symptoms are assessed by the ambulance service using the “Face – Arm – Speech – Time” test (FAST) or the prehospital National Institutes of Health Stroke Scale (NIHSS) [33, 34]. After clinical assessment, all patients with suspected stroke symptoms are directly consulted with the on-call stroke physician at OUS. If not accepted for hospitalization these patients are transported to their local hospital or to an out-of-hours GP acute clinic for further diagnostic follow-up [23].

### Participants

We recruited participants among dispatchers at EMCC Oslo, Norway, aiming to achieve a purposeful sampling of information-rich participants [27] who represented the diversity of employees at EMCC Oslo, in terms of education, age, sex and working experience as medical dispatchers. Participants were recruited both through verbal invitations during an annual training course, and a post on the internal website of the EMCC. The department management approved the time spent on interviews as working hours, which were accepted as compensatory time off. No further compensation was provided to participants.

### Research team and reflexivity

The first author (BJ) is a male intensive care nurse with work experience from the ambulance services in Oslo, Intensive care unit at Oslo University Hospital and the Norwegian Board of Health Supervision. Since 2013 he has held a position as a senior adviser at the Norwegian Directorate of Health. He has no clinical or personal relationship with the participants.

The co-authors are medical doctors and nurses representing a wide range of expertise on pre-hospital emergency medicine, stroke care, qualitative methods, and medical research. One of the co-authors also holds a part-time position as a medical dispatcher at EMCC Oslo.

The study follows the consolidated criteria for reporting qualitative studies (COREQ) [35]. The COREQ checklist is available in Supplemental materials.

### Data collection

The first author (BJ) conducted in-depth, semi-structured interviews based on an interview guide. The interview guide was developed by the first and last authors (BJ and CH) and was based on results from previous research [23, 36–41]. The interview guide addressed three main topics; contextual and environmental conditions, such as workload, staffing, physical environment, factors that hinder or facilitate stroke recognition, and experiences related to assessments based on the decision support tool in use (Index). All questions were open-ended, intended to create room for new, unexpected phenomena [42]. When appropriate, the interviewer also asked follow-up questions and rephrased the participants’ answers to ensure accurate comprehension. The interview guide was pilot-tested with one medical dispatcher and adjustments were made based on the experiences gained. The pilot interview was included in the analysis as it only led to minor changes in the interview guide. The interview guide is available in Supplemental Materials.

The interviews were conducted during August and October 2022. Twelve interviews were conducted face to face at the Norwegian National Advisory Unit on Prehospital Emergency Medicine, Oslo University Hospital. Three interviews were conducted digitally as video calls using Microsoft Teams © Video Conferencing System. All interviews included a presentation of the interviewer and the aims of the study. All interviews were digitally recorded, anonymized, and subsequently transcribed verbatim by an external transcriber and the first author. After each interview, the interviewer wrote down initial impressions and thoughts from the interview as reflexivity notes. Interviews were continued until saturation was reached, indicated by data replication, and the absence of new information emerging, as discussed among the team [27].

Fifteen medical dispatchers expressed their interest in participating in the study. All were invited to participate, and none withdrew from the study after being interviewed. No repeat interviews were conducted, and transcripts were not returned to the participants for comments. Additionally, no feedback routines were established for the findings.

Among the participants, 13 (87%) were female, compared to 70% females in the overall group of medical dispatchers. Six dispatchers had prehospital working experience from the ambulance services or out-of-hours general practitioners (GP) acute clinics. Six dispatchers had prior work experience from in-hospital emergency departments or intensive care units. Only one dispatcher had working experience from a stroke unit. In median, the participants had been working as medical dispatchers at EMCC Oslo for two years. Baseline characteristics of participants are presented in Table 1.

**Table 1:** Baseline characteristics of participants

Total number of participants *n*=	15
Female *n*=	13
Age, median years (range)	32 (26–48)
Nurses *n*=	13
Paramedics *n*=	2
Prehospital working experience *n*=	6
In-hospital emergency department working experience *n*=	6
Working experience at EMCC Oslo, median years (range)	2 (0.5–7)

The median length of interviews were 53 min (range 33–82 min) resulting in 49 written pages. A total of 1184 meaning-bearing units/initial codes were identified. The codes were sorted into 16 initial themes, which were then refined into four final themes.

### Data analysis

Interviews were analyzed using a stepwise thematic analysis in-line with recommendations from Braun & Clarke [43]. Hyper Research © version 4.0, was used to facilitate the analysis process. The analysis process included the following six steps:

Step one: The interviewer (BJ) thoroughly reviewed all transcripts and reflexivity notes from the interviews to become acquainted with the data, gain an overall understanding of the content, and identify the most interesting findings.

Step two: The first and last authors (BJ, CH) separately identified meaning-bearing units (initial codes) from the transcripts representing different aspects of the participants’ experiences and thoughts.

Step three: Initial codes were sorted into initial themes by the first author. The initial themes were subject of repeated discussions between the first, second and last authors (BJ, EJS, CH).

Step four: The themes were discussed with all co-authors in relation to the essence of each theme and the aim of the study. As a result, the initial themes were revised.

Step five: Based on repeated discussions between the first, second and last authors (BJ, EJS, CH), we reached consensus on definition and naming final themes.

Step six: Based on the fully worked-out themes we presented and discussed the results relevant to the research question.

An example of initial codes, initial and final themes are presented in Table 2.

Quotations presented in the Results section are numbered consecutively with corresponding references to the various participants. (Quote 1 (# 14) = Quote number 1, participant number 14)

**Table 2:** Example of initial codes, initial and final themes

Initial codes	Initial themes	Final themes
We are forced to make quick decisions due to the frequent breaches of EMCC response time interval requirements. Whether I collect information about the patient’s medical history and regular medications depends on the current workload. Given the high work pressure, our priority is to conclude calls as quickly as possible after confirming the ABCs.	Gathering patient information about their medical history, medication, and possible risk factors is considered important for assessing suspected stroke calls. Due to the high workload, dispatchers are compelled to restrict the duration of the emergency calls.	Contextual conditions at EMCC can affect dispatcher’s decision-making process and the ability for experiential learning.
If an index criterion is met, it legitimizes the dispatch of an ambulance. I often end up dispatching an ambulance for situations that I don’t really think require one. If an index criterion is met, I don’t care how little or how much I believe in it or how realistic it seems.	If an index criterion is met, ambulances are typically dispatched. Ambulances are dispatched even when the dispatcher, based on a professional assessment, believes there is no need for an ambulance.	Consistent use of the CBD protocol may increase EMCC overtriage.

### Ethical considerations

The Regional Committees for Medical Research Ethics (REC) Southeast Norway (ref. no. 2018/1909) and the local data protection officer at Oslo University Hospital (ref. no. 18/25297) approved the study. The study was performed according to the principles stated in the Declaration of Helsinki [44] and was based on informed, voluntary, and written informed consent to participate, including the right to withdraw from the study at any time. We informed all participants about the principles of anonymity and confidentiality concerning publication of the results from the interviews. We emphasized that the researchers would not exchange information with the department management regarding individuals’ performances or answers.

## Results

All medical dispatchers interviewed perceived stroke as a time-critical condition in which the EMCC played an important role in identifying stroke symptoms, thus contributing to reducing prehospital delay and improving patient outcomes. Additionally, we identified four final themes that affect medical dispatchers’ decision-making in stroke calls:

1. Pronounced stroke symptoms are easy to identify.
2. Non-specific neurological symptoms raise suspicion of acute stroke but are difficult to differentiate from other medical conditions.
3. Consistent use of the CBD protocol may increase EMCC overtriage.
4. Contextual conditions at EMCC can affect dispatcher’s decision-making process and the ability for experiential learning.

### Theme 1. Pronounced stroke symptoms are easy to identify

The dispatchers consistently report that pronounced stroke symptoms consistent with the Face-Arm-Speech-Time (FAST) test, always raise suspicion of acute stroke and are easy to identify. Dispatchers also regard a stroke as likely if the caller expresses concern that the patient is experiencing a stroke.

#### Quote 1 (# 14)

*If the caller says the patient suddenly lost strength in his arm or has become lopsided in his face*,* I suspect a stroke.*

#### Quote 2 (# 13)

*It takes quite a bit to miss the signs of a stroke if there are typical stroke criteria.*

### Theme 2. Non-specific neurological symptoms raise suspicion of acute stroke but are difficult to differentiate from other medical conditions

A substantial number of dispatchers reported that they suspect a stroke if the caller describes the patient as *suddenly dizzy*, *have symptoms they have not experienced before*, *feels* or *behaves strangely*, *is not themselves*, *became confused* or *experienced sudden and intense headache*. Nevertheless, most dispatchers find it challenging to determine whether such non-specific neurological symptoms represent stroke symptoms or symptoms of other medical conditions. They experience this as a particular challenge when assessing patients with sequelae from previous strokes, multiple concurrent symptoms, as well as elderly patients with multiple comorbidities and/or dementia.

#### Quote 3 (# 11)

*What often makes me suspect a stroke is that the caller explains that the patient has become clumsy or is not acting like himself.*

#### Quote 4 (# 4)

*Sudden confusion can have multiple causes. Often*,* it affects elderly. In such cases*,* it is difficult to determine whether it is cerebral dysfunction or an infection.*

We identified various opinions among dispatchers regarding the utility of the EMCC dispatch protocol in use for stroke assessments. Nevertheless, most dispatchers consider the dispatch protocol in use as a useful tool for assessing patients with pronounced stroke symptoms consistent with the FAST criteria, but numerous dispatchers report limited utility for assessing patients with more vague and non-specific stroke symptoms.

#### Quote 5 (# 12)

*I think the dispatch protocol is a good decision support tool for stroke patients*,* but I find it challenging to assess dizziness and unsteadiness when there are no FAST symptoms.*

Several dispatchers reported that they perceived sudden onset of symptoms as decisive for suspecting a stroke, both among patients with pronounced stroke symptoms and patients with more vague and non-specific stroke symptoms. Although the dispatchers were not explicitly asked about their assessment of persistent or fluctuating stroke symptoms, none of them reported considering such symptoms as decisive for suspecting a stroke.

#### Quote 6 (# 9)

*Stroke patients with diffuse symptoms have in common that they usually describe the symptoms as having occurred quite suddenly. Not something*,* that has developed very gradually.*

### Theme 3. Consistent use of the dispatch protocol may increase EMCC overtriage

All dispatchers consider the dispatch protocol as mandatory for assessing and prioritizing emergency calls. Many dispatchers also report that they apply the dispatch protocol consistently and to the letter, leaving little room for triage based on their own professional expertise. Consequently, ambulances are dispatched even when the professional assessment suggests that there is no need for an ambulance. Several dispatchers report that this consistent use of the dispatch protocol obviously increases EMCC overtriage. The consistent use of the dispatch protocol is partly explained by dispatchers training, as well as serving as a safeguard against individual complaints and/or supervisory cases.

#### Quote 7 (# 8)

*I’ve learned that the dispatch protocol is a procedure to be followed.*

#### Quote 8 (# 9)

*Complaints and supervisory cases naturally influence the job*,* and we were already informed during training that you will encounter a complaint case at some point.*

#### Quote 9 (# 6)

*We are always told that if we follow the dispatch protocol*,* we are within acceptable limits. So*,* in a way*,* it becomes your lifeline.*

Most dispatchers also report that overtriage is considered an acceptable approach to prevent undertriage. Furthermore, they reported that overtriage rarely had been addressed or problematized, neither by the dispatchers themselves, nor by the department management.

#### Quote 10 (# 8)

*As my supervisor said*,* you don’t get any credit for not dispatching an ambulance.*

#### Quote 11 (# 10)

*I’m not concerned about overtriage. I think we can handle those false dispatches. It’s about not taking any chances.*

### Theme 4. Contextual conditions at EMCC can affect dispatcher’s decision-making process and the ability for experiential learning

All dispatchers consistently reported an exceptionally high workload and insufficient staffing situation at the EMCC, which frequently led to multiple queued emergency calls and breaches of response time requirements. Consequently, dispatchers feel compelled to restrict the duration of the emergency calls.

Even though some dispatchers claim that assessment of emergency calls solely should be based on current patient symptoms, the majority of dispatchers regard a patient’s medical history, medication, and other risk factors as highly pertinent for assessing suspected stroke calls in general, but especially in cases of doubt. Nevertheless, the capacity to obtain such patient information is perceived as restricted by the heavy workload and the necessity to meet EMCC response time interval requirements.

#### Quote 12 (# 1)

*We are forced to make quick decisions due to the frequent breaches of EMCC response time interval requirements.*

#### Quote 13 (# 8)

*When you are under time pressure*,* the questioning becomes more symptom-based.*

Another consequence of the high workload is that dispatchers seldom can consult with colleagues or the on-call EMCC emergency physician in cases of doubt.

#### Quote 14 (# 3)

*It’s very hectic. Very often*,* you just have to make a decision yourself. You don’t have the opportunity to confer with a colleague because everyone is busy.*

The dispatchers also report that the high workload rarely enables formal and informal professional discussions or debriefings.

Most dispatchers emphasize that experiential learning is crucial for enhancing their own assessments of emergency calls. However, there is no established system for experiential learning or feedback loops in place at the EMCC in question.

#### Quote 17 (# 12)

*I believe there’s a lot of learning in feedback loops*,* but we don’t have any such system.*

#### Quote 18 (# 7)

*It’s very rare that I get to know what the patient’s problem actually was.*

#### Quote 19 (# 13)

*You become blind to yourself and end up in the usual routine*,* doing things the way you’ve always done.*

Dispatchers also reported that, until recently, the EMCC had an annual recertification process for all dispatchers, which involved a review of actual emergency calls. This allowed dispatchers to receive feedback on how they handled a number of their emergency calls. However, due to the high workload and the need to train new employees, the recertification process and the review of emergency calls have not been conducted recently.

## Discussion

In this study, we found that medical dispatchers consistently perceive stroke as a time-critical medical condition, in which the EMCC plays a key role in reducing prehospital delays and improving patient outcomes. The dispatchers reported that pronounced stroke symptoms are easy to assess and triage. Conversely, assessing non-specific stroke symptoms such as dizziness, confusion or changed behaviour is considered challenging to differentiate from symptoms of other less time-critical medical conditions. They also reported that the high EMCC workload restricts the ability to collect pertinent patient information and consult with their colleagues in cases of doubt. Furthermore, the EMCC lacks feedback loops which restricts their ability to obtain experiential learning.

Decision-making in clinical medical practice is a complex process influenced by various factors including the availability of and access to evidence-based guidelines, assessment skills and contextual factors such as resource availability, legal requirements, and interaction with other healthcare professionals. It is also shaped by external pressures such as patient demands and media influence [45–48].

Unlike most other clinical settings, decision-making at EMCC poses a particular challenge due to the inability to visually observe and physically examine the patient. As a result, communication skills and the interaction between the caller and the medical dispatcher must be considered particularly important in the decision-making process at the EMCCs [24, 49]. Furthermore, the assessment of medical response and priority also relies on two additional components: the dispatcher’s medical competence and the content and application of dispatch protocols [50].

In this study we found that EMCC medical dispatchers consider pronounced stroke symptoms consistent with the Face-Arm-Speech-Time test (FAST) as easy to identify. This finding aligns with results from other published studies [7, 39, 51], as well as the findings from our first study at EMCC Oslo, Norway, which indicated that 87% of the stroke patients identified at the EMCC (EMCC true positive stroke patients) had pronounced stroke symptoms consistent with the FAST-test [23].

Conversely, we found that most medical dispatchers perceive vague and non-specific stroke symptoms, such as dizziness, confusion, or altered behaviour, challenging to differentiate from symptoms of other, and less time-critical medical conditions. Additionally, many dispatchers perceive the current CBD protocol as less supportive in assessing such symptoms.

Given that medical dispatchers perceive stroke as a time-critical condition where EMCC plays a crucial role in improving outcomes, these findings suggest that assessing vague and non-specific stroke symptoms, independent of other factors, could increase EMCC overtriage.

Another finding was that many dispatchers report consistent use of the CBD dispatch protocol, leaving limited room for triage based on their professional expertise. This finding indicates that medically trained dispatchers, to some extent, use the current CBD dispatch protocol in a manner similar to how algorithm-driven (MPD) dispatch protocols are used by medical untrained dispatchers [52]. This deviates from the intended use of this type of decision support tool and could obviously increase EMCC overtriage. Interestingly, medical dispatchers explained that the consistent use of the dispatch protocol serves as a safeguard against individual complaints and supervisory cases. This type of conservative approach leading to medical overtreatment is well-known from other healthcare services [53].

Several medical dispatchers also reported that overtriage is seldom addressed, neither by the dispatchers, nor by the department management. This finding also aligns with the aims and findings of many other studies on EMCC accuracy, which primarily focus on enhancing sensitivity and with less emphasis on addressing the unintended consequences of overtriage [54]. It is also reasonable to assume that lack of specific guidelines for acceptable levels of overtriage in EMCCs could exacerbate the acceptance of high levels of overtriage [22]. The fact that overtriage is not addressed by department management may indicate not only a lack of clear expectations regarding medical dispatchers’ performance but also the absence of mutually agreed quality objectives for the EMCC service. This finding is considered particularly interesting, partly because over the past five years, we have experienced a 27% increase in the number of inquiries to EMCC and a simultaneous 23% increase in the number of high-priority ambulance missions nationally. Additionally, Norway’s publicly funded healthcare system is experiencing a growing shortage of qualified healthcare personnel, making it necessary to reduce unnecessary overtriage [55, 56].

Based on several media reports prior to the interviews, we had a preconception about the high workload at the EMCC in question. This preconception was confirmed by all medical dispatchers and aligns with the results from Leonardsen et al. who found that lack of resources and managerial support could impact EMCC accuracy negatively [57]. Nevertheless, our study revealed several interesting consequences associated with the high EMCC workload.

A key finding was that the high workload and strict EMCC response time interval requirements, which stipulate that 90% of all emergency calls to the EMCC should be answered within 10 seconds, made the dispatchers feel compelled to limit the duration of emergency calls. This, in turn, restricted their ability to gather essential patient information such as medical history, medications, and other risk factors before making decisions on dispatch priority. The limited gathering of such essential patient information clearly hampers EMCC accuracy and may lead to both unintentional undertriage and overtriage. The findings from our initial study at EMCC Oslo, Norway, emphasize the risk of undertriage, revealing that the median assessment time interval for stroke patients not identified by the EMCC was only 55 s [23].

Medical dispatchers also reported that the high workload diminishes their ability to seek guidance from colleagues or the on-call EMCC emergency physician in case of doubt, as well as the ability to engage in formal and informal discussions and debriefings. Although establishing causality between the lack of guidance and mis-triage is difficult, results from other studies report frequent reliance on colleagues’ assistance in dealing with difficult calls and a tendency to dispatch ambulances more frequently in cases of doubt [2, 58, 59].

Experiential learning, defined as learning derived from the effective utilization of current experiences [60], relies on the existence of feedback loops and other kinds of reflective practice. These processes offer valuable insights, whether positive or negative, into the effects of interventions such as medical assessments, and are regarded as an essential characteristic for the development of professional competence among a variety of health professions [61].

In our study, medical dispatchers reported a lack of systems for feedback loops or other strategies for experiential learning within the EMCC. Consequently, dispatchers had limited opportunities to assess and improve their performance on daily basis. Moreover, it is also reasonable to assume that discontinuing the annual recertification routines for medical dispatchers has exacerbated the negative impact of insufficient experiential learning practices [62].

In this qualitative study, we identified several contextual conditions affecting medical dispatchers’ decision-making in suspected stroke calls, subsequently reducing the accuracy of EMCC assessments. Our findings also underscore the necessity to enhance the accuracy of EMCC assessments for patients with less pronounced stroke symptoms. Future efforts to distinguish less pronounced stroke symptoms from other less acute medical conditions in EMCCs could clearly benefit from the utilization of artificial intelligence (AI) as a decision support tool. Consequently, we argue that future efforts should encompass both the development and implementation of AI, alongside further research, and enhancement of contextual conditions.

### Strengths and limitations

We succeeded in achieving a purposeful sampling of information-rich participants [27], who could be representative of the diversity of dispatchers at EMCC Oslo, both in terms of education, age, sex and working experience as medical dispatchers. However, we cannot rule out that the recruitment of participants, to some extent, may have influenced the results.

The varied backgrounds within the research team have enriched the interpretations of the data and contributed to critical discussions and reflections on both the content and connection between the data, and how the researchers’ preconceptions could influence the interpretations [23].

The external validity of this study is limited by the fact that we only interviewed employees from one Norwegian EMCC. However, having reached saturation with our sample, we feel confident about our findings regarding this EMCC that coordinated ambulance dispatches during our initial study at EMCC Oslo [23]. The EMCC in question used a CBD protocol, which is less common than algorithm-based dispatch systems. Nevertheless, results from other published studies using both algorithm-based and CBD protocols [22, 23], report substantial variations in both sensitivity and specificity. Consequently, we argue that our findings, highlighting how factors such as high workload, EMCC response time requirements, and the lack of experiential learning practices impact the decision-making process of dispatchers, could likely be generalized to other EMCCs, regardless of the specific dispatch protocols in use.

## Conclusion

Medical dispatchers perceive vague and non-specific stroke symptoms, such as dizziness, confusion or altered behaviour, challenging to differentiate from symptoms of other and less time-critical medical conditions. They also perceive the current CBD protocol in use as less supportive in assessing such symptoms.

High workload and strict EMCC response time interval requirements hinder the gathering of essential patient information and the ability to seek guidance in cases of doubt, potentially exacerbating both EMCC undertriage and overtriage.

The absence of feedback loops and other strategies for experiential learning in the EMCC hampers the medical dispatcher’s ability to evaluate their own assessments and improve dispatch accuracy.

In conclusion, the findings from this study suggest that future initiatives to enhance EMCC accuracy in identifying suspected stroke patients should include the development and implementation of artificial intelligence (AI) to support assessments of patients with less pronounced stroke symptoms. Additionally, it emphasizes the need for further research and improvement of contextual conditions to optimize EMCC performance.

## Electronic supplementary material

Below is the link to the electronic supplementary material.

Supplementary Material 1

## Data Availability

The unidentified datasets generated during the analyses of the current study are available from the corresponding author on reasonable request.

## Abbreviations

AIS: Acute Ischemic Stroke
AMIS: Acute Medical Information System
CBD: Criteria Based Dispatch
Dispatch NASPP: The Dispatch –Norwegian Acute Stroke Prehospital Project
ED: Emergency department
EMCC: Emergency Medical Communication Centre
EMD: Emergency Medical Dispatcher
EMS: Emergency Medical Services
FAST: Face Arm Speech Test
GP: General Practitioner
HEMS: Helicopter Emergency Medical Services
ICH: Intracerebral Hemorrhage
Index: The Norwegian Index for Emergency Medical Assistance
MSU: Mobile Stroke Unit
NIHSS: National Institutes of Health Stroke Scale
OUS: Oslo University Hospital Ullevål
ParaNASPP: Paramedic Norwegian Acute Stroke Prehospital Project
PPV: Positive Predictive Value
REC: Regional Research Ethics Committee
TIA: Transient Ischemic Attack
TSD: Service for Sensitive Data
