# Supplementary material for: Factors affecting emergency medical dispatchers decision making in stroke calls – a qualitative study
Source: BMC Emerg Med. 2024 Nov 15;24:214. doi: 10.1186/s12873-024-01129-0 (PMC11566283; doi:10.1186/s12873-024-01129-0)
Supplement: Supplementary file 1 — Supplementary Material 1 [file 12873_2024_1129_MOESM1_ESM.pdf]

Additional materials

Supplementary illustration 1

The Norwegian Index for Emergency Medical Assistance, 3rd edition, Index Start card

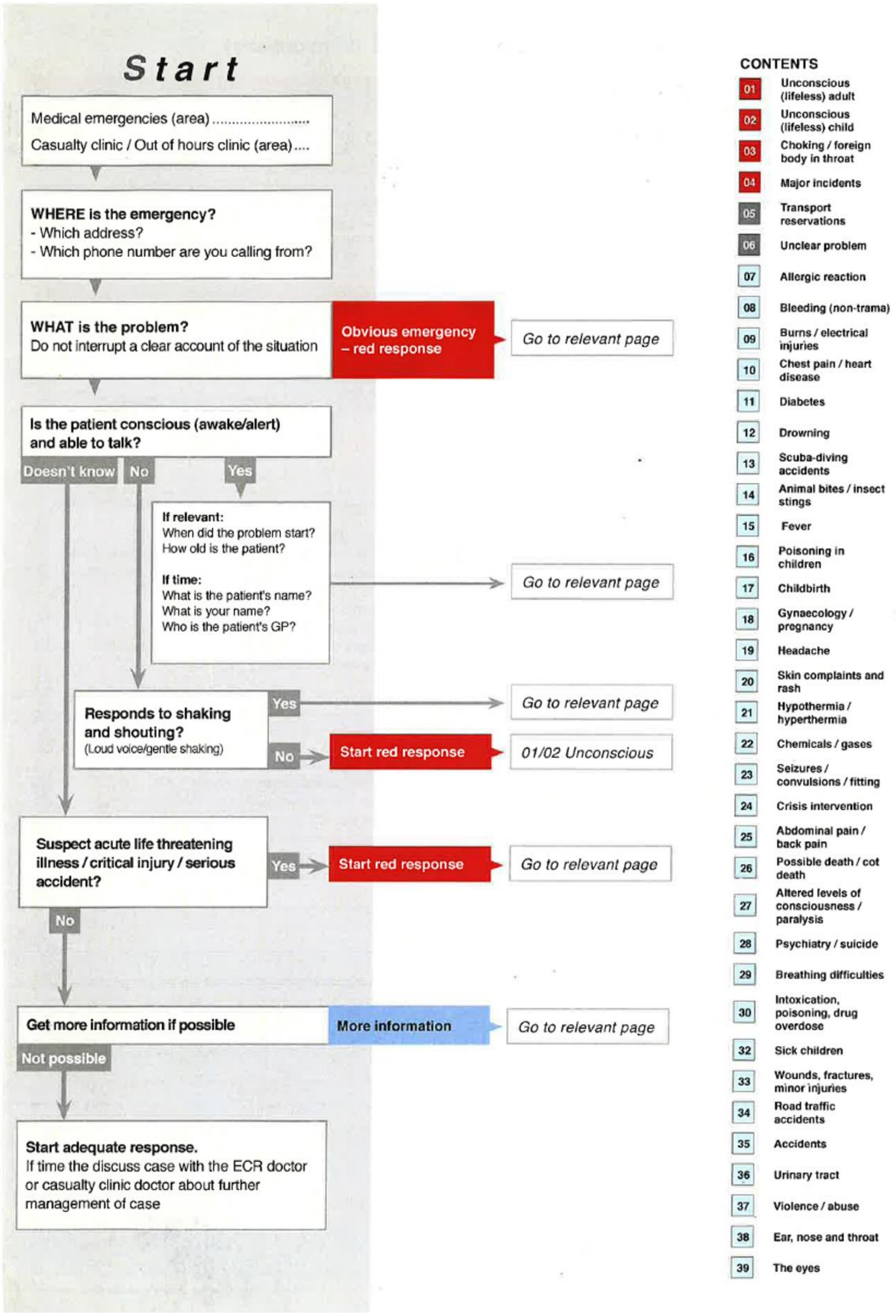

Supplementary illustration 2

The Norwegian Index for Emergency Medical Assistance, 3rd edition, criteria card 27.

27 Altered levels of consciousness / paralysis

1 CRITERIA / ADVICE

Bleeding: *See 08 Bleeding (non-trauma)*
Diabetes: *See 11 Diabetes*
Convulsions: *See 23 Convulsions/fits*
Ilntoxication, poisoning, drugs: *See 30 Intoxication, poisoning, drug ove*
Sick child: *See 32 Sick children*
Injuries: *See 35 Accidents*

Priority	Code	Criteria	Advice
Priority I	A.27.01	**Does not respond to shaking and shouting.**	6
Priority I	A.27.02	**Breathing difficulties.**	1.4.5.10
Priority I	A.27.03	**Suddenly lop-sided (asymmetrical, irregular) in face.**	2.4.5
Priority I	A.27.04	**Sudden loss of strength in an arm or a foot.**	2.4.5
Priority I	A.27.05	**Sudden speech difficulties.**	2.4.5
Priority I	A.27.06	**Increasingly confused / drowsy, suspect stroke.**	1.4.5
Priority I	A.27.07	**Sudden onset of intense and unfamiliar headache.**	1.2.4.5
Priority I	A.27.08	**Still conscious (awake/alert), feels faint.**	1.3.4.5
Priority I	A.27.09	**Pale and clammy.**	1.3.4.5
Priority I	A.27.10	**Sudden onset of abdominal pain.**	3.4.5.9
Priority I	A.27.11	**Sudden rapid pulse, seems weak.**	3.4.5
Priority II	H.27.01	**Epileptic, has had a fit, still unconscious (lifeless).**	1.4.5.8
Priority II	H.27.02	**Has had a fit, still confused / drowsy after 20 minutes.**	1.4.5.8
Priority II	H.27.03	**Suddenly confused / drowsy for no known reason.**	1.3.4.5
Priority II	H.27.04	**Prolonged confusion / drowsiness.**	1.4.5
Priority II	H.27.05	**Sudden paralysis rapidly reverting to normal again.**	2.4.5
Priority II	H.27.06	**Lost consciousness (fainted) several times within last 24 hours.**	4.5
Priority II	H.27.07	**Sudden loss of vision in one eye.**	2.4.5
Priority III	V.27.01	**Fainted, OK now.**	5
Priority III	V.27.02	**Fainted after taking glyceryl trinitrate, awake now.**	2.5
Priority III	V.27.03	**Has epilepsy, waking up gradually following a fit.**	8

** There may be an indication for rapid thrombolytic treatment if signs and symptoms indicate possible stroke, even if the symptoms started several hours ago. If it is less than 4 – 5 hours since the onset of symptoms: Ask relevant additional questions and immediately contact the doctor on call at the nearest stroke unit (or other unit that offers thrombolytic treatment) for advice on level of urgency.*

2 RESPONSE

Casualty clinic
1. Keep in touch with caller.
2. Alert ambulance / doctor on call, or as prearranged with the ECR.
3. Contact ECR and assist with local knowledge.

ECR
1. Alert ambulance / doctor on call.
2. If relevant assess also:
   - Alerting nearest ambulance (even with patient).
   - Alerting nearest doctor (even if not on call).
   - Alerting air ambulance, mobile doctor, specialist ambulance.
   - Suggest transport in private vehicle.
3. Check the remaining criteria.
4. Ask relevant additional questions.
5. Give appropriate advice to the caller.
6. Keep in touch with caller if possible.
7. Get more information from the casualty clinic / doctor on call if relevant.
8. Monitor the situation and update all units (including casualty clinic).
9. Offer hints to health personnel.

Local adjustments:

Date ____ Signed (doctor) ____

Casualty clinic / ECR
1. Check the remaining criteria.
2. Ask relevant additional questions.
3. Give appropriate advice to caller.
4. Do one of the following according to local protocols.
   - Connect caller to doctor on call.
   - Inform doctor on call and contact caller again.
   - Send an ambulance.
   - Ask patient to come to the casualty clinic / outpatients.
5. Ask caller to phone back immediately if condition worsens.

ECR
1. Inform the casualty clinic.

Casualty clinic
1. Ask relevant additional questions.
2. Give appropriate advice to caller.
3. Follow local protocols e.g.:
   - Inform doctor on call.
   - Ask patient to phone the doctor on call at ...... (time).
   - Ask the patient to come to the casualty centre at ...... (time).
   - Arrange further telephone contact with the caller.
   - Inform the community nurse.
   - Ask the patient to call his/her GP on the next working day.
   - Inform patient's GP on the next working day.
   - End the call if there is clearly no need for help.
4. Ask caller to phone back immediately if condition worsens.

ECR
1. Do one of the following according to local protocols e.g.:
   - Pass on to casualty clinic.
   - Ask the caller to phone the casualty clinic.

3 ADDITIONAL QUESTIONS

This episode
Any discomfort beforehand? Nausea?
Vomiting? Temperature?
Breathing normally? Superficially? Deeply?
Labouring?
Has patient had a fit? Incontinent?
Possible overdose? Tablets? Drugs? Alcohol?

Earlier episodes
Has patient had anything similar before? What was the reason then?
Has patient had a stroke or a TIA (transient ischaemic attack) before?
Is patient on daily medication? What?
Does patient suffer from any particular illness?
Heart disease?
Epilepsy?
High blood pressure?
No-one knows patient
Does patient have a medic-alert bracelet or badge?

Suspect stroke
- Exactly when did the symptoms start? (Important if less than 4½ hours ago).
- Has s/he had a fit? When, how long did it last?
- Is s/he awake/alert? Can you wake him/her?
- Does s/he use blood thinning tablets (anti-coagulants like Warfarin, Plavix, Aspirin?)
- Was s/he self reliant before this?
- Has s/he had a stroke before?
- Has s/he had brain surgery?

Degree of semi-consciousness
Can patient speak normally? Disoriented?
Single words? Incomprehensible sounds?
Can patient move his/her arms and legs if you ask?
Open eyes?
Does patient open his/her eyes if you shake him/her

4 ADVICE TO CALLER

1. **Altereds level of consciousness (sleepiness, drowsiness) or breathing difficulties**
   Make sure s/he can breathe freely.
   Loosen tight clothing.
   If s/he can't sit up, lie patient on his/her side. Tilt the head back carefully and lift the chin forwards. (Children under 1 year: Keep the head in a normal position and lift chin forwards). Make sure s/he is still breathing.
2. This may be the onset of a stroke and should be assessed immediately by a doctor. Lie the patient in a comfortable position, preferably with the upper part of the body elevated.
3. **If patient stops breathing, start CPR**
   See 01/02 Unconscious adult/child.
4. **Find any medication s/he uses.**
   Have it ready for the ambulance personnel.
5. **Do not leave patient unattended.**
   Report any deterioration immediately.
6. See 01/02 Unconscious adult/child.
7. See 10 Chest pain / heart disease.
8. See 23 Seizures / convulsions / fitting.
9. See 25 Abdominal pain / back pain.
10. See 29 Breathing difficulties.

5 HINTS FOR HEALTH PERSONNEL

I can put you in touch with a hospital specialist. Assess possible differential diagnoses that require specific treatment.

Assess level of consciousness
using Glasgow Recovery Scale.

Verbal response	Eye opening
5. Oriented	4. Spontaneous
4. Confused	3. Opens eyes to speech
3. Random words	2. Opens eyes to pain
2. Incomprehensible noises	1. No eye opening
1. No verbal response	

Motor response
6. Obeys commands
5. Localises pain
4. Flexion withdrawal
3. Abnormal flexion
2. Abnormal extension
1. No response

The Glasgow recovery scale is the sum of these three parametres mentioned above.

Stroke.
Oxygen: 7-10 l. / min. by mask or 2-3 l. / min. in nasal catheter.
Ensure an open airway.
Ambulance transport with head elevated, or recovery position.
Prehospital treatment of hypertension is usually contraindicated.
If thrombolytic treatment not relevant:
- Assess acetylsalicylates orally as quickly as possible in consultation with hospital dept.
- If less than 2 hours since onset of symptoms: Assess immediate admission (red response) for thrombolytic treatment in consultation with relevant dept.

About altered levels of consciousness / paralysis

SYNCOPE
A sudden drop in blood pressure due to a passing decrease in the blood supply to the brain can lead to brief loss of consciousness. Common reasons for fainting are: Intense pain, psychological stress, sudden change of position from lying to standing, ingestion of Glyceryl trinitrate, passing urine whilst standing. The patient will regain consciousness within 2 mins. while the body is horizontal and the mechanism of physiological compensation increases the blood pressure. Injuries incurred on falling are often more serious than the reason for fainting.

STROKE (APOPLEXY)
A stroke is usually due to a blockage in one of the cerebral arteries (thrombosis or embolism). This results in infarction. Strokes can also occur when there is spontaneous bleeding into the tissues of the brain. Symptoms vary depending on the extent of the injury, and the area of the brain affected. Paralysis or numbness in one side of the body or face, speech difficulties, difficulty swallowing, vision disturbance, and varying degrees of consciousness are commonly seen. Deep coma and abnormal respiration are serious signs.

TRANSIENT ISCHAEMIC ATTACK
Stroke symptoms with total recovery within 24 hours. Usually due to an embolism from one of the major arteries or the inside of the heart which get wedged in one of the smaller arteries of the brain and affect the blood supply. The attack can be a precursor to a stroke and should be checked immediately by a specialist.

SUBARACHNOIDAL HAEMORRHAGE
Is due to sudden haemorrhaging from a dilatation (aneurysm) in one of the cerebral arteries. Bleeding may start spontaneously, or can be triggered off by hypertension in connection with physical exertion. Typical signs: the patient will suffer from a sudden and intense headache often described as the worst ever experienced. Other symptoms can include neck stiffness, nausea, vomiting, confusion, reduced level of consciousness, paralysis, speech difficulties and convulsions. The patient can lose consciousness and stop breathing. The condition is very serious and often affects young people without any warning.

EMERGENCY TREATMENT OF STROKE
Stroke patients were not given priority earlier. However, good results from active treatment have changed attitudes. All stroke units now offer immediate assessment and the start of thrombolytic treatment in the same way that cardiac units do for heart attacks. The time span for when thrombolysis for stroke is beneficial, and the indications and counter indications, are being constantly reassessed. If it is less than 4 ½ hours since symptoms started, thrombolytic treatment should be considered. Ask additional questions to clarify the situation. Confer with the neurologist on call at the local stroke unit, or other unit offering treatment, and leave the final decision about whether there are indications for thrombolytic treatment to the doctor. The response must be red / emergency in that case. The patient must be transported rapidly to hospital (air ambulance should be considered) for final assessment with cerebral CAT scan.

RAISED INTRACEREBRAL PRESSURE
Reduced levels of consciousness after head injury may be due to epidural or subdural haematoma (haemorrhaging in the tissue of the brain), or to cerebral oedema. The condition rapidly becomes critical if cerebral hernia threatens. Other reasons for raised intracranial pressure can be spontaneous haemorrhaging into the cranium, a brain tumour or a cerebral shunt that is blocked and not draining off the cerebrospinal fluid (shunt failure). If pressure rises rapidly, the condition becomes critical with cerebral hernia threatening.

ACUTE ARRHYTHMIAS
The most common forms of arrythmia that lead to semi-consciousness or loss of consciousness are ventricular tachycardia or a sudden attack of Stokes Adams syndrome.

ACUTE INTERNAL HAEMORRHAGING
Acute fall in blood pressure due to internal haemorrhaging can cause loss of consciousness. Most commonly dissecting aortic aneurysm, bleeding into the intestine or into the abdominal cavity from the liver, the spleen, or the kidneys, or an ectopic pregnancy (See 08 Bleeding non-trauma).

OTHER REASONS FOR SUDDEN LOSS OF CONSCIOSNESS
- Febrile convulsions: see 23 Seizures, convulsions and fitting
- Post-convulsive state: see 23 Seizures, convulsions and fitting
- Hypoglycaemia in diabetics: See 11 Diabetes
- Intoxication, overdose and poisoning: see 30 Intoxication, poisoning, drug overdose
- Hyperventilation: See 28 Psychiatry
- Hysterical psychogenic reaction: See 28 Psychiatry
- Heatstroke: See 21 Hypothermia/hyperthermia

Supplementary illustration 3

The Norwegian Index for Emergency Medical Assistance, 3rd edition, criteria card 39

39 The eyes

1 Criteria

Priority	Code	Criteria	Advice
Priority I	A.39.01	Sudden vision disturbance, feels faint.	2
Priority I	A.39.02	Large open injury to or near the eye.	1
Priority I	A.39.03	Blow to the eye / head. Still conscious (awake/alert) but feeling faint.	1
Priority I	A.39.04	Extensive corrosives injury to or near the eye.	4
Priority I	A.39.05	Ingested methanol, impaired vision.	8.9
Priority I	A.39.06	Sudden loss of vision in one eye.	2
Priority II	H.39.01	Severe continuous pain in the eye, feels unwell.	6.7.8
Priority II	H.39.02	Gradual onset of reduced vision in one or both eyes for a couple of days.	7
Priority II	H.39.03	Moderate corrosives injury in or near eye.	4.8
Priority II	H.39.04	Bruising (bluish) round both eyes after head injury.	1.8
Priority II	H.39.05	Ingested methanol, vision still normal.	8.9
Priority II	H.39.06	Red, painful and irritated eye.	7
Priority II	H.39.07	Stinging / painful rash round one eye.	7
Priority III	V.39.01	Strong or continuous pain in or near eye, otherwise OK.	6.7.8
Priority III	V.39.02	Speck of something stuck in eye.	5.8
Priority III	V.39.03	Red and irritable eye.	3.8
Priority III	V.39.04	Red eye without irritation or pain.	8
Priority III	V.39.05	Has been in strong sunlight. Pain and impaired vision.	6.8
Priority III	V.39.06	Has been welding. Pain and impaired vision.	6.8
Priority III	V.39.07	Black eye (bruising), no other symptoms.	8

2 Response

Casualty clinic
1. Keep in touch with caller.
2. Alert ambulance / doctor on call, or as prearranged with the ECR.
3. Contact ECR and assist with local knowledge.

ECR
1. Alert ambulance / doctor on call.
2. If relevant assess also:
   -Alerting nearest ambulance (even with patient).
   -Alerting nearest doctor (even if not on call).
   -Alerting air ambulance, mobile doctor, specialist ambulance.
   -Suggest transport in private vehicle.

3. Check the remaining criteria.
4. Ask relevant additional questions.
5. Give appropriate advice to the caller.
6. Keep in touch with caller if possible.
7. Get more information from the casualty clinic / doctor on call if relevant.
8. Monitor the situation and update all units (including casualty clinic).
9. Offer hints to health personnel.

Local adjustments:

Date ____ Signed (doctor) ____

Casualty clinic / ECR
1. Check the remaining criteria.
2. Ask relevant additional questions.
3. Give appropriate advice to caller.

4. Do one of the following according to local protocols.
   -Connect caller to doctor on call.
   -Inform doctor on call and contact caller again.
   -Send an ambulance.
   -Ask patient to come to the casualty clinic / outpatients.

5. Ask caller to phone back immediately if condition worsens.

ECR
1. Inform the casualty clinic.

Casualty clinic
1. Ask relevant additional questions.
2. Give appropriate advice to caller.
3. Follow local protocols e.g.:
   -Inform doctor on call.
   -Ask patient to phone the doctor on call at ...... (time).
   -Ask the patient to come to the casualty centre at ...... (time).
   -Arrange further telephone contact with the caller.
   -Inform the community nurse.
   -Ask the patient to call his/her GP on the next working day.
   -Inform patient's GP on the next working day.
   -End the call if there is clearly no need for help.
4. Ask caller to phone back immediately if condition worsens.

ECR
1. Do one of the following according to local protocols e.g.:
   -Pass on to casualty clinic.
   -Ask the caller to phone the casualty clinic.

Supplementary illustration 4

COREQ Checklist

COREQ (COnsolidated criteria for REporting Qualitative research) Checklist

A checklist of items that should be included in reports of qualitative research. You must report the page number in your manuscript where you consider each of the items listed in this checklist. If you have not included this information, either revise your manuscript accordingly before submitting or note N/A.

Topic	Item No.	Guide Questions/Description	Reported on Page No.
**Domain 1: Research team and reflexivity**			
Personal characteristics			
Interviewer/facilitator	1	Which author/s conducted the interview or focus group?	4
Credentials	2	What were the researcher's credentials? E.g. PhD, MD	3
Occupation	3	What was their occupation at the time of the study?	3
Gender	4	Was the researcher male or female?	3
Experience and training	5	What experience or training did the researcher have?	3
Relationship with participants			
Relationship established	6	Was a relationship established prior to study commencement?	3
Participant knowledge of the interviewer	7	What did the participants know about the researcher? e.g. personal goals, reasons for doing the research	24
Interviewer characteristics	8	What characteristics were reported about the inter viewer/facilitator? e.g. Bias, assumptions, reasons and interests in the research topic	24
**Domain 2: Study design**			
Theoretical framework			
Methodological orientation and Theory	9	What methodological orientation was stated to underpin the study? e.g. grounded theory, discourse analysis, ethnography, phenomenology, content analysis	6
Participant selection			
Sampling	10	How were participants selected? e.g. purposive, convenience, consecutive, snowball	5
Method of approach	11	How were participants approached? e.g. face-to-face, telephone, mail, email	5
Sample size	12	How many participants were in the study?	5
Non-participation	13	How many people refused to participate or dropped out? Reasons?	5
Setting			
Setting of data collection	14	Where was the data collected? e.g. home, clinic, workplace	5
Presence of non-participants	15	Was anyone else present besides the participants and researchers?	5
Description of sample	16	What are the important characteristics of the sample? e.g. demographic data, date	5
Data collection			
Interview guide	17	Were questions, prompts, guides provided by the authors? Was it pilot tested?	5
Repeat interviews	18	Were repeat inter views carried out? If yes, how many?	5
Audio/visual recording	19	Did the research use audio or visual recording to collect the data?	5
Field notes	20	Were field notes made during and/or after the inter view or focus group?	5
Duration	21	What was the duration of the inter views or focus group?	6
Data saturation	22	Was data saturation discussed?	5
Transcripts returned	23	Were transcripts returned to participants for comment and/or	5

Topic	Item No.	Guide Questions/Description	Reported on Page No.
		correction?	
**Domain 3: analysis and findings**			
Data analysis			
Number of data coders	24	How many data coders coded the data?	6
Description of the coding tree	25	Did authors provide a description of the coding tree?	6
Derivation of themes	26	Were themes identified in advance or derived from the data?	6
Software	27	What software, if applicable, was used to manage the data?	6
Participant checking	28	Did participants provide feedback on the findings?	5
Reporting			
Quotations presented	29	Were participant quotations presented to illustrate the themes/findings? Was each quotation identified? e.g. participant number	6
Data and findings consistent	30	Was there consistency between the data presented and the findings?	6
Clarity of major themes	31	Were major themes clearly presented in the findings?	6
Clarity of minor themes	32	Is there a description of diverse cases or discussion of minor themes?	6

Developed from: Tong A, Sainsbury P, Craig J. Consolidated criteria for reporting qualitative research (COREQ): a 32-item checklist for interviews and focus groups. *International Journal for Quality in Health Care*. 2007. Volume 19, Number 6: pp. 349 – 357

Once you have completed this checklist, please save a copy and upload it as part of your submission. DO NOT include this checklist as part of the main manuscript document. It must be uploaded as a separate file.

Supplementary illustration 5

Interview-guide

Topics	Research questions	Interview questions
Introduction		Presentation of the interviewer and completion of form regarding personal information about medical dispatchers who were interviewed.<br><br>Introduction including a brief presentation of the Dispatch NASPP project and focus for the interview.<br><br>• Main target is to improve EMCC stroke recognition at a system – and not individual level<br><br>• Different methodological approaches; a quantitative study based on EMCC- and hospital records, medical dispatcher interviews, and analysis of EMCC audio files<br><br>• Results from the interviews will be presented in such a way that the interviewees cannot be identified<br><br>• The researchers will not have any dialogue with the department management about individuals' performances or answers to our questions<br><br>• In the interviews, we are looking for the operators' spontaneous personal/subjective assessments and opinions.<br><br>• The interviews will include the following topics:<br><br>o Brief presentation of yourself as an medical dispatcher (separate form)<br>o Reflections on EMCC contextual conditions<br>o How you suspect stroke during emergency calls<br>o What facilitates and what complicates recognition of stroke patients<br>o Your use of Norwegian Index for Emergency Medical Assistance as a dispatch decision support tool<br>o Other relevant issues<br><br>• Methods<br>Semi-structured in-depth interview<br>The interview will last no more than 2 hours.<br>We will perform audio recordings that are transcribed verbatim<br>We haven't planned a review or approval of the interview transcript - but a review will be possible on request

Topics	Research questions	Interview questions
Contextual conditions	How contextual conditions, and psychosocial conditions could affect medical dispatchers performance	First I would like to hear about how you experience to work as a medical dispatcher at EMCC Oslo<br><br>• Could you for instance describe a typical working shift at EMCC Oslo<br><br>• How do you perceive<br>o Staffing and workload<br>o Your opinion of the quality of care provided at EMCC<br>o The atmosphere at work<br>o Break processing during shifts<br>o Is it common to have discussions/dialogue with other medical dispatchers about current events<br>o Do you have any feedback loops to find out if own assessments were correct<br>o What's the opportunities of professional development as a medical dispatcher<br><br>Could you describe the division of labor and cooperation between medical dispatchers, resource coordinators, operations manager, on-call EMCC physician, and Helicopter Emergency Medical Services-coordinator<br><br>How does this affect your work as a medical dispatcher?<br><br>Could you describe your own motivation in relation to your job at EMCC Oslo – for instance<br>o Why did you apply for a job as an medical dispatcher at EMCC Oslo<br>o Is the job as expected?<br>o How do you enjoy work?<br>o Do you feel safe at work as a medical dispatcher?<br>o Do you think that patient complaints or supervisory cases against EMCC and/or medical dispatcher affects your performance as a medical dispatcher? If Yes – how?<br>o Any other issues that could influence your performance as a medical dispatcher?

Topics	Research questions	Interview questions
Emergency calls and recognition of suspected stroke symptoms	Knowledge of stroke and stroke patient treatment<br><br>Knowledge of symptoms and risk factors related to stroke.<br><br>Factors affecting stroke patient outcome<br><br>The medical dispatcher role in the chain of stroke survival<br><br>How do the dispatchers understand and interpret callers' descriptions of stroke symptoms?<br><br>What factors hinder or facilitate the identification of stroke and the dispatching of necessary resources?	Now I want to talk a little bit more specifically about recognizing patients with possible stroke symptoms.<br><br>We want to emphasize that this is in no way a knowledge test. We would therefore appreciate that your give us your own and spontaneous reflections on the topics we present to you.<br><br>First of all; what kind of training have you received on recognition of stroke patients?<br>• New employee course?<br>• Annual training courses<br><br>Why do you think it is important to recognize stroke patients at EMCC?<br><br>What aspects of the dialogue with the caller lead you to initially suspect that the patient may have a stroke?<br><br>What do you consider important in order to recognize patients with possible stroke symptoms?<br><br>Are there any specific patient details that could strengthen the suspicion of a stroke?<br><br>If you suspect a stroke, do you conduct or structure the conversation in a particular way?<br><br>When it comes to urgency in suspected stroke cases, what are your thoughts?<br><br>Is it important to spend time questioning or quickly dispatch the ambulance and ending the call?<br><br>How do you collaborate/interact with others when suspecting a stroke:<br>• Resource coordinator<br>• Operations manager<br>• Other medical operators<br>• Ambulance personell

<table>
<tr><th>Topics</th><th>Research questions</th><th>Interview questions</th></tr>
<tr><td></td><td></td><td>Do you usually update AMIS information about the patient if you continue the conversation with the caller after dispatching the ambulance?

What makes it difficult to recognize patients with stroke?

If in doubt whether the patient may have a stroke, can you seek advice from others:
• Other medical dispatchers
• Operations manager
• On call EMCC physician
• On call Air ambulance physician or stroke physician

Do you personally consider that you have sufficient knowledge about stroke symptoms?</td></tr>
<tr><td>The use of the Norwegian Index for Medical Emergency Assistance</td><td>Factors influencing the use of the dispatch protocol in general and specifically in handling emergency calls concerning patients with possible stroke symptoms.</td><td>In general how do you use the dispatch protocol
• Always
• Occasionally
• If you don't recognize the patient's condition or know what advice to give
• Mandatory or voluntary use of the index

Do you mostly use the dispatch protocol to assess criteria and urgency or to provide instructions to the caller?
As a trained healthcare professional, what are your thoughts on using the dispatch protocol compared to relying on your own healthcare/medical expertise?

How do you experience the dispatch protocol as a decision support tool for recognizing stroke patients?

Which criteria cards do you think are most relevant for assessing patients with suspected stroke?

27: Altered consciousness-paralysis
19: Headache
39: Eye
06: Unresolved problem

How do you experience the dispatch protocol functioning when you suspect stroke?

Do you have any other procedures or routines that may be helpful in assessing whether a patient has a stroke?</td></tr>
</table>

Topics	Research questions	Interview questions
		Have you experienced a lack of relevant criteria for assessing stroke patients in the dispatch protocol, and if so, which ones?<br><br>How do you proceed if there is a need to provide feedback or suggestions for improvements to the content of the dispatch protocol?
	Other important considerations.	The last - but often the most important - question always comes at the end, namely:<br><br>What have I forgotten to ask you about that you think affects your ability to recognize patients with probable stroke?

Supplementary illustration 6

Registration form regarding medical dispatchers who were interviewed.

Name:
Internal EMCC ID:
Age:
Sex: Male ☐ Female ☐ Other ☐
Education: Nurse ☐ EMT ☐ Paramedic ☐
Other:
Specialization:
Position: Full time ☐ Part time ☐ Proportion:
Position as a medical dispatcher at EMCC Oslo since:
Have you been working in another EMCC ?:
Any other work experience from prehospital emergency services:
Any work experience from in-hospital emergency departments:
Do you have work experience from in-hospital stroke units?

YES /NO If YES – from where:
Were you employed as a medical dispatcher at EMCC Oslo during the period from September 1st 2019, until February 29th 2020?
YES/NO
Role/function at EMCC Oslo:
Medical dispatcher ☐ Resource Coordinator ☐ Operations manager ☐
Professional developer ☐ Other role/function:
